# lin28 proteins promote expression of 17∼92 family miRNAs during amphibian development

**DOI:** 10.1002/dvdy.24358

**Published:** 2015-11-14

**Authors:** Fiona Warrander, Laura Faas, Oleg Kovalevskiy, Daniel Peters, Mark Coles, Alfred A. Antson, Paul Genever, Harry V. Isaacs

**Affiliations:** ^1^ Department of BiologyUniversity of YorkYorkYO10 5DDUK; ^2^ York Structural Biology Laboratory, Department of Chemistry, University of YorkHeslington YorkYO10 5DDUK; ^3^ Centre for Immunology and Infection, University of YorkHeslington YorkYO10 5DDUK

**Keywords:** lin28, mir‐363, let‐7, mir‐17∼92, mir‐106∼363, *Xenopus*

## Abstract

**Background:** Lin28 proteins are post‐transcriptional regulators of gene expression with multiple roles in development and the regulation of pluripotency in stem cells. Much attention has focussed on Lin28 proteins as negative regulators of *let‐7* miRNA biogenesis; a function that is conserved in several animal groups and in multiple processes. However, there is increasing evidence that Lin28 proteins have additional roles, distinct from regulation of *let‐7* abundance. We have previously demonstrated that lin28 proteins have functions associated with the regulation of early cell lineage specification in *Xenopus* embryos, independent of a lin28/*let‐7* regulatory axis. However, the nature of lin28 targets in *Xenopus* development remains obscure. **Results:** Here, we show that mir‐17∼92 and mir‐106∼363 cluster miRNAs are down‐regulated in response to lin28 knockdown, and RNAs from these clusters are co‐expressed with lin28 genes during germ layer specification. Mature miRNAs derived from *pre‐mir‐363* are most sensitive to lin28 inhibition. We demonstrate that lin28a binds to the terminal loop of *pre‐mir‐363* with an affinity similar to that of *let‐7*, and that this high affinity interaction requires to conserved a GGAG motif. **Conclusions:** Our data suggest a novel function for amphibian lin28 proteins as positive regulators of mir‐17∼92 family miRNAs. *Developmental Dynamics 245:34–46, 2016*. © 2015 Wiley Periodicals, Inc.

## Key Findings

- We show that mir‐17∼92 and mir‐106∼363 cluster miRNAs are down regulated in response to lin28 knockdown in Xenopus embryos.
- We demonstrate that lin28a binds to the terminal loop of pre‐mir‐363 and this interaction requires a conserved a GGAG motif.
- Our data suggest a novel function for amphibian lin28 proteins as positive regulators of mir‐17∼92 family miRNAs.

## Introduction

### Lin28 Proteins Are Posttranscriptional Regulators

Lin28 family proteins are posttranscriptional regulators of development and adult homeostasis. They are RNA binding proteins, characterised by a unique combination of RNA binding cold shock and zinc knuckle domains. LIN‐28 was initially identified as a regulator of developmental timing in *C. elegans* and is required for the self‐renewal of stem cells, with mutations in LIN‐28 leading to the precocious development of late cell lineages (Moss et al., 1997; Vadla et al., 2012). Mammalian embryonic stem cells also express high levels of Lin28 proteins, which, in combination with Nanog, Oct4 and Sox2, have been used to reprogram somatic cells to a pluripotent stem cell phenotype (Viswanathan and Daley, 2010). Lin28 family genes have also been implicated as regulators in a diverse range of other biological processes, including glucose homeostasis, tissue regeneration, and the onset of puberty in both mice and humans (Shyh‐Chang and Daley, 2013; Shyh‐Chang et al., 2013).

Research in several different systems has focused on the conserved role of Lin28 proteins as negative regulators of *let‐7* family miRNAs. Lin28 proteins interact with both primary and precursor *let‐7* miRNAs to inhibit the biogenesis of the mature biologically active forms. A prevalent model indicates an inverse relationship between levels of Lin28 proteins and mature *let‐7* miRNAs (Viswanathan, 2008; Viswanathan and Daley, 2010). Typically, reduction in Lin28 function leads to increased levels of mature *let‐7* miRNAs. This regulatory interaction between Lin28 proteins and *let‐7* miRNAs is clearly important in multiple contexts, however, there is also increasing evidence for interactions of Lin28 proteins with a wider range of RNA targets, including other miRNA families and multiple protein coding mRNAs (Mayr and Heinemann, 2013). In the latter situation, interaction with Lin28 proteins has been shown to affect the translation of the target mRNA (Mayr and Heinemann, 2013; Shyh‐Chang and Daley, 2013).

### Lin28 Function in Amphibian Development

In an earlier study, we identified *lin28a* as a transcriptional target of FGF signalling (Branney et al., 2009). Subsequently, we investigated the function of the two Lin28‐related genes, *lin28a* and *lin28b*, in *Xenopus* (Faas et al., 2013). We showed that compound knockdown of lin28a and lin28b in early development disrupts the development of axial and paraxial mesoderm. Our data indicate that lin28 function is required in pluripotent cells of the early *Xenopus* embryo for the normal response to mesoderm inducing growth factors signals, such as FGF and activin.

### Identifying miRNA Targets of Amphibian lin28 Proteins

At present, the nature of lin28 target RNAs in the early amphibian embryo remains elusive. Our data show that *Xenopus* lin28a and lin28b are able to interact with the terminal loop of *let‐7* miRNAs (Faas et al., 2013). However, inhibition of lin28 function in *Xenopus* does not lead to significant increases in the levels of mature *let‐7* miRNAs in the early embryo. Therefore, the earliest perturbations in amphibian development, resulting from lin28 knockdown, do not arise from effects on a lin28/*let‐7* axis (Faas et al., 2013).

In the present study, we have undertaken a microarray based analysis to determine if other miRNAs are regulated by lin28 in gastrula stage amphibian embryos. In contrast to the prevailing model, in which Lin28 proteins act as negative regulators of miRNA biogenesis, we find that lin28 knockdown leads to significant down‐regulation of several miRNAs. Prominent amongst these are *mir‐363‐5p* and *mir‐363‐3p*, which are derived from a common *mir‐363* precursor RNA.

*mir‐363* belongs to the mir‐17∼92 family of miRNAs, which are encoded by the mir‐17∼92, mir‐106a∼363, and mir‐106b∼25 genomic clusters. These paralogous clusters are transcribed to produce polycistronic RNAs, which are subsequently processed to form multiple, mature miRNAs with a range of related seed sequences and target specificities (Olive et al., 2010; Mogilyansky and Rigoutsos, 2013). Significantly, we find that several other miRNAs from both the mir‐17∼92 and mir‐106a∼363 clusters are also down‐regulated in response to lin28 inhibition, indicating that *Xenopus* lin28 proteins may have a wider role in regulating the abundance mir‐17∼92 family miRNAs.

We demonstrate that zygotic transcription of the mir‐17∼92 and mir‐106a∼363 clusters is initiated in the *Xenopus* embryo during the period of germ layer specification. We show that *mir‐363‐5p* and *mir‐363‐3p* are both expressed in the early mesoderm and later in the neuroectoderm in domains overlapping with those previously reported for *lin28a* and *lin28b* (Faas et al., 2013), suggesting a possible role for a lin28/mir‐17∼92 regulatory axis in the process of germ layer specification.

The mechanism by which lin28 proteins regulate the abundance of mir‐17∼92 family miRNAs remains unclear; however, we show here that lin28a protein physically interacts with a GGAG motif in the in the terminal loop of the *pre‐mir‐363* miRNA. Our data support a novel function for *Xenopus* lin28 proteins as positive regulators of *mir‐363* miRNA abundance.

## Results

### Analysis of miRNA Abundance in lin28 Morphant Embryos

**Figure 1:**
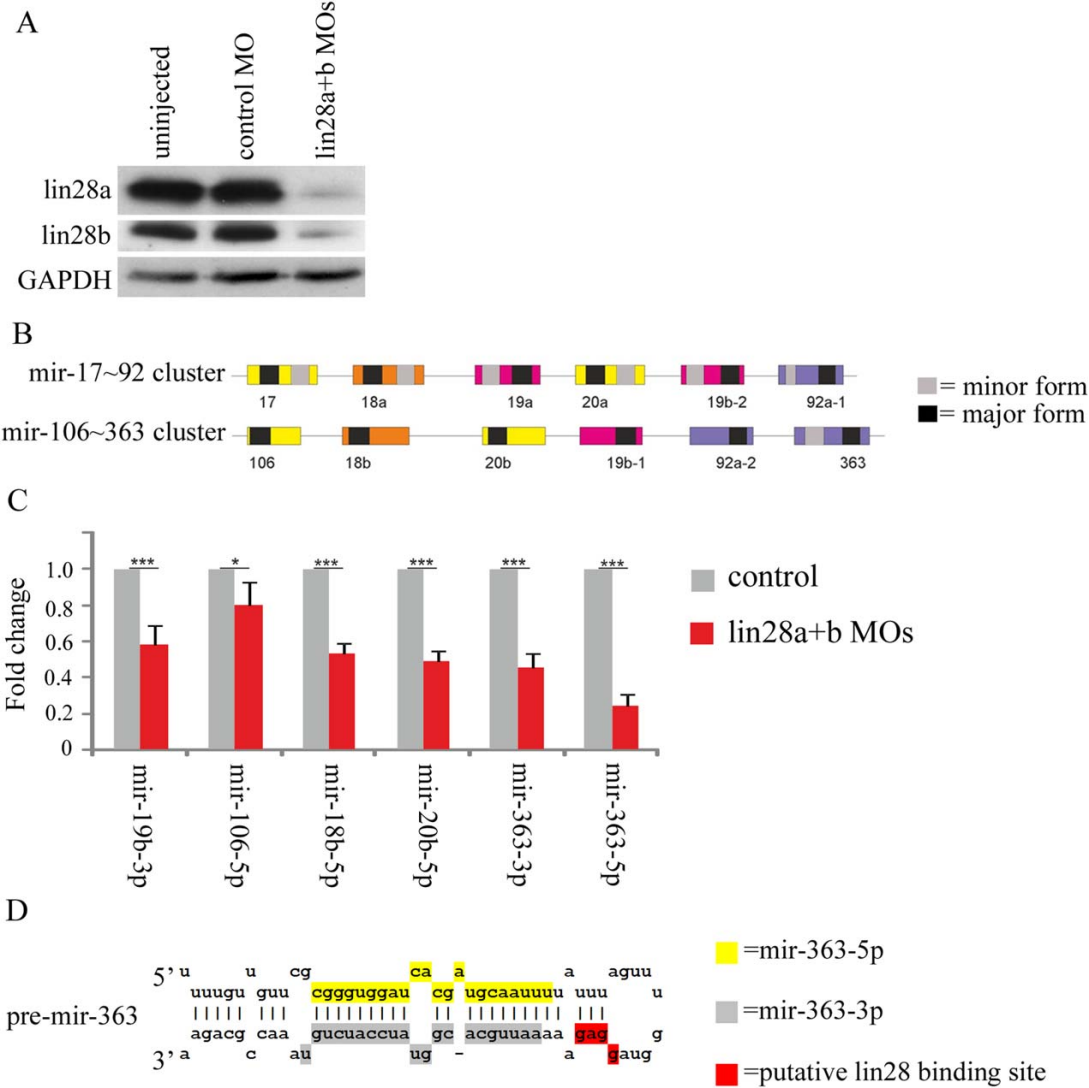
**A**: Western blot analysis of endogenous lin28a and lin28b expression in embryos injected with a total of 12.5 ng/embryo of lin28 MOs in the compound knockdown compared with CMO‐injected and uninjected control embryos at stage 16. GAPDH was used as a loading control. **B**: Scale diagram showing the genomic organisation of *Xenopus* mir‐17∼92 and mir‐106∼363 clusters. Coloured boxes indicate pre‐miRNA sequences, with each colour corresponding to paralog groupings based on seed sequence. Where known, the black and grey boxes, respectively, represent the major and minor forms derived from a common precursor. **C**: qRT‐PCR was performed on embryos injected with 10 ng each/embryo of lin28a1, a2 and b MOs and control embryos, at stage 10.5. Fold change in expression of miRNAs is shown compared with controls and normalised using U6 by the 2^‐ΔΔCt^ method. Fold change is given as average of 3 biological replicates, with error bars representing SE. **D**: Predicted secondary structure of *Xenopus pre‐mir‐363*. The sequences of mature *mir‐363‐5p, mir‐363‐3p*, and the putative lin28 binding site are indicated.

We have previously reported the efficient knockdown of endogenous *Xenopus* lin28 proteins using a combination of antisense morpholino oligos (AMOs) directed against the three lin28 isoforms (lin28a1, lin28a2, and lin28b) expressed in the embryo. In contrast to the predictions of the prevailing model for Lin28 function, we found no evidence for change in *let‐7* abundance at gastrula stages following lin28 knockdown (Faas et al., 2013). This begs the question, are there other miRNA targets of lin28 proteins in the earliest stages of amphibian development? To begin to address this question, we have used the same AMOs to efficiently knockdown endogenous lin28 proteins (Fig. 1A) and microarray analyses to identify changes in the abundance of miRNAs in lin28 knockdown embryos (lin28 morphants) at two different stages (early gastrula stage 10.5 and late gastrula stage 13). These screens were carried out using the Affymetrix microarray platform at stage 10.5 and the Exiqon microarray platform at stage 13 (Supp. Tables S1 and S2, which are available online).

An analysis of fold changes relative to controls of *Xenopus* miRNAs with an expression level of threshold >10 in control and lin28 morphant embryos at early gastrula stage 10.5 reveals several miRNAs changing in abundance. However, using a strict cut‐off of ≥ two‐fold change and *P* ≤ 0.05, we find that only one miRNA (*mir‐363‐5p*) is significantly affected in lin28 morphants, in this case showing a 2.6‐fold decrease, (*P* = 0.049), indicating a positive role for lin28 in regulating the abundance of this miRNA.

At gastrula stage 13, more miRNAs are affected in Lin28 morphants. Table 1 shows the fold changes of *Xenopus* miRNA abundance in lin28 morphants relative to control embryos at stage 13. Only miRNAs flagged as being detected in all replicate arrays were included in this table. We find that several miRNAs show significant (≥two‐fold change and *P* ≤ 0.05) changes in abundance in stage 13 lin28 morphants, including *mir‐363‐3p*, which, like *mir‐363‐5p*, is processed from a common *mir‐363* precursor RNA. The *mir‐363* precursor is derived from a polycistronic primary RNA which is transcribed from the mir‐106a∼363 locus of clustered miRNAs; a paralogue of the well characterised mir‐17∼92 miRNA cluster. Figure 1B shows the organisation of the *X. tropicalis* mir‐17∼92 and mir‐16∼363 loci as derived from the *X. tropicalis* genome sequence. As indicated in Table 1, six of eight of the significantly changing miRNAs are transcribed from these clusters, with the abundance of all decreasing in lin28 morphants.

**Table 1:** Changes in miRNA Expression in Late Gastrula Stage 13 lin28 Morphant Embryos^a^

miRNA	Mean control	Mean AMO	Fold change in morphant relative to control	Member of mir‐106∼363 cluster	Member of mir‐17∼92 cluster
*xtr‐miR‐20a*	*1.50*	*0.51*	*−2.9*		***X***
*xtr‐miR‐17‐5p*	*1.26*	*0.52*	*−2.4*		***X***
*xtr‐miR‐200a*	*1.25*	*0.59*	*−2.1*		
*xtr‐miR‐20b*	*1.29*	*0.61*	*−2.1*	***X***	
xtr‐miR‐301	1.14	0.54	−2.1		
*xtr‐miR‐363‐3p*	*1.28*	*0.60*	*−2.1*	***X***	
*xtr‐miR‐19a*	*1.36*	*0.67*	*−2.0*		***X***
*xtr‐miR‐19b*	*1.27*	*0.63*	*−2.0*	***X***	
*xtr‐miR‐428*	*1.27*	*0.63*	*−2.0*		
xtr‐miR‐200b	1.37	0.71	−1.9		
xtr‐miR‐130b	1.32	0.86	−1.5		
xtr‐miR‐203	1.14	0.75	−1.5		
xtr‐miR‐30b	1.13	0.90	−1.3		
xtr‐miR‐125a	1.27	1.11	−1.1		
xtr‐miR‐126	2.46	2.43	−1.0		
xtr‐miR‐427	1.09	1.04	−1.0		
xtr‐let‐7c	1.10	1.20	−0.9		
xtr‐let‐7a	1.16	1.14	1.0		
xtr‐let‐7e	0.97	0.99	1.0		
xtr‐miR‐155	1.09	1.15	1.1		
xtr‐miR‐22	0.92	1.00	1.1		
xtr‐miR‐7	0.93	1.27	1.4		

a The expression of *Xenopus* miRNAs in lin28 morphants and control embryos at late gastrula stage 13. Expression levels are shown as ratios relative to abundance in a mixed stage reference RNA sample. Only miRNAs flagged as being detected in all three replicate arrays are included. miRNAs showing ≥ 2 fold change and *P* ≤ 0.05 are shown in italic type. Memberships of mir‐106∼363 and mir‐17∼92 clusters are indicated.

We next investigated quantitative changes in the levels of several mir‐17∼19 and mir‐106a∼363 cluster miRNAs in lin28 morphants by quantitative real‐time polymerase chain reaction (qRT‐PCR). Again, we see significant decreases in the abundance of several 17∼92 family miRNAs (Fig. 1C), including *mir‐363‐3p* and *mir‐363‐5p*. There is good evidence that GGAG or closely related motifs in the terminal loop regions of pre‐miRNAs are important for recognition and binding by the zinc knuckle domain of lin28 proteins (Mayr and Heinemann, 2013). Here we show that such a GGAG is present in the *Xenopus mir‐363* precursor RNA. Figure 1D indicates the putative lin28 binding motif and highlights the predicted sequences of the mature *mir‐363‐5p* and *mir‐363‐3p* miRNAs.

### Analysis of miRNA Abundance in lin28 Over‐expressing Embryos

**Figure 2:**
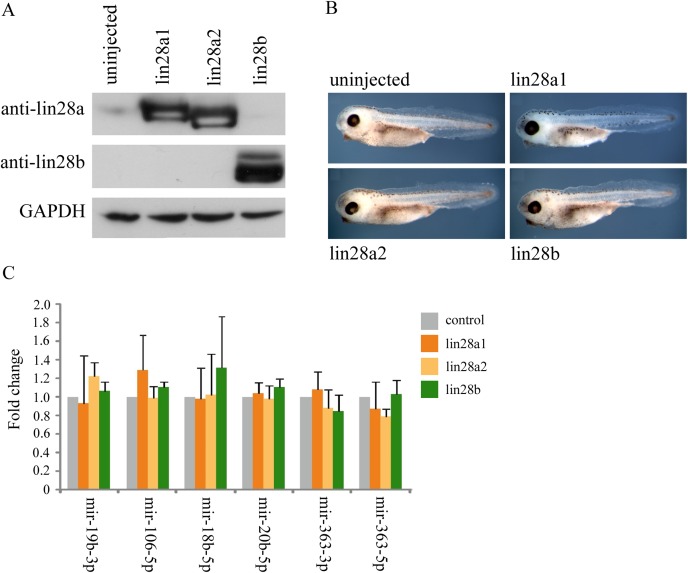
**A**: Western blot analysis of lin28 proteins at stage 10.5 in control embryos and embryos injected with 1 ng of mRNAs coding for either lin28a1, lin28a2 or lin28b. GAPDH was used as a loading control. **B**: Phenotype of control embryos and embryos overexpressing either lin28a1, lin28a2, or lin28b at stage 38. **C**: qRT‐PCR was performed on embryos injected with control embryos and either 1 ng of mRNAs coding for lin28a1, a2 or b MOs at stage 10.5. Fold change in expression of miRNAs is shown compared with controls and normalised using U6 by the 2^‐ΔΔCt^ method. Fold change is given as average of 3 biological replicates, with error bars representing SE.

We were interested to see how over‐expression of the three *Xenopus* lin28 proteins affected embryo development and the abundance of 17∼92 family miRNAs. Figure 2A is a Western blot showing levels of each protein in embryos injected with 1ng of each of the synthetic lin28 mRNAs. In contrast to the Western blot in Figure 1A, the exposure presented does not detect endogenous lin28 proteins in control uninjected embryos, indicating that mRNA injection leads to massive overexpression of each of the lin28 proteins relative to normal endogenous levels. Interestingly, overexpression of lin28 proteins does not result in gross developmental abnormalities (Fig. 2B) or significant changes in the abundance of mature mir‐17∼19 and mir‐106a∼363 cluster miRNAs (Fig. 2C).

### Physical Interaction of Recombinant lin28a Protein With the mir‐363 Terminal Loop

**Figure 3:**
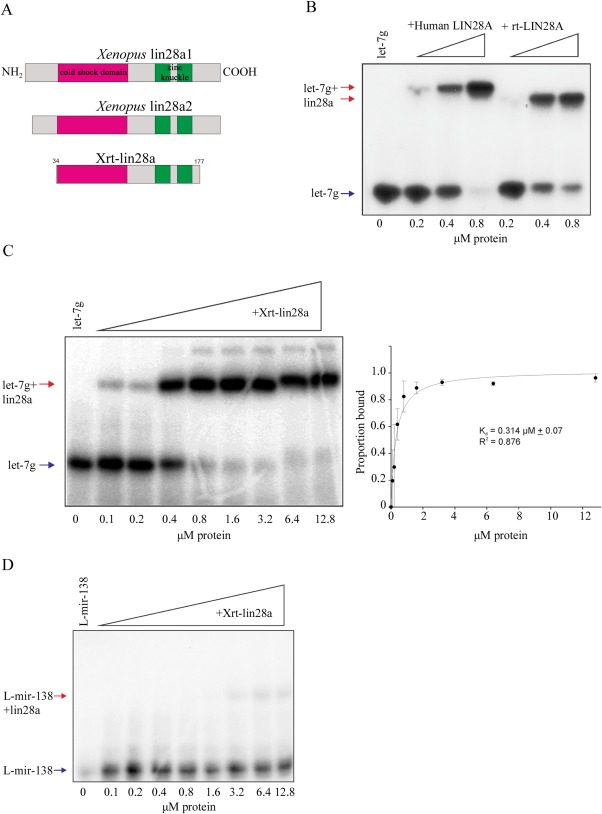
**A**: Scale diagram of the *Xenopus* proteins used in this study. Cold shock domains are shaded magenta and zinc knuckles green. **B**: EMSA performed with ^32^P‐labelled L‐let‐7g and indicated concentrations of human recombinant LIN28A protein, either full‐length or truncated (rt). Arrows indicate labelled RNA (blue) and LIN28A‐RNA complex (red). **C**: EMSA performed with ^32^P‐labelled L‐let‐7g and indicated concentrations of Xrt‐lin28a. Gel shown is representative of n = 3. Arrows indicate labelled RNA (blue) and lin28‐RNA complex (red). Band intensities were quantified from three independent experiments and the proportion bound was calculated. Data were fit by nonlinear regression as described in Materials and Methods. B_max_ = 1.017. **D**: EMSA performed with ^32^P‐labelled L‐mir‐138 and indicated concentrations of Xrt‐lin28a. Arrows indicate RNA and lin28a‐RNA complex. Gel shown is representative of n = 3. Arrows indicate labelled RNA (blue) and lin28‐RNA complex (red).

As with lin28 regulation of *let‐7* miRNAs, it is possible that lin28 regulation of *mir‐363* abundance involves a physical interaction of the protein with the miRNA terminal loop. We used RNA electromobility shift assays (EMSAs) to investigate whether *Xenopus* lin28 proteins bind to *pre‐mir‐363* RNA. We purified a recombinant, truncated version of the *X. tropicalis* lin28a (Xrt‐lin28a). This protein construct of residues 34‐177, contains both the cold shock and zinc‐knuckle RNA binding domains, but lacks segments of N‐terminal and C‐ terminal residues, which were predicted to be disordered (Fig. 3A). To determine if terminal truncations affect binding to canonical RNA targets, we compared the abilities of recombinant, full‐length human and N‐ and C‐ terminally truncated human LIN28A (rt‐LIN28A, residues 37‐180) to bind the terminal loop of a *let‐7‐g* precursor RNA; a well characterised lin28 target, which we have previously shown to be bound by in vivo translated *Xenopus* lin28 proteins (Faas et al., 2013). Figure 3B shows that full‐length and truncated human LIN28A proteins have a similar ability to bind *let‐7g* RNA. In keeping with these observations, we find that that Xrt‐lin28a is also able to bind the *let‐7g* terminal loop with high affinity (K_d_ = 314 nM) (Fig. 3C). We provide additional evidence that Xrt‐lin28a maintains its ability to discriminate genuine target RNAs. *Pre‐mir‐138* has previously been shown to be ineffective at competing with pre‐let‐7 for binding of Lin28a protein, indicating that the terminal loop of *mir‐138* is not a high affinity target of Lin28 proteins (Piskounova et al., 2008). Figure 3D shows that Xrt‐lin28a protein also exhibits little binding activity toward the terminal loop of *mir‐138* (L‐mir‐138). Even at the highest protein concentrations tested, the proportion of radiolabelled L‐mir‐138 bound is only 22%.

**Figure 4:**
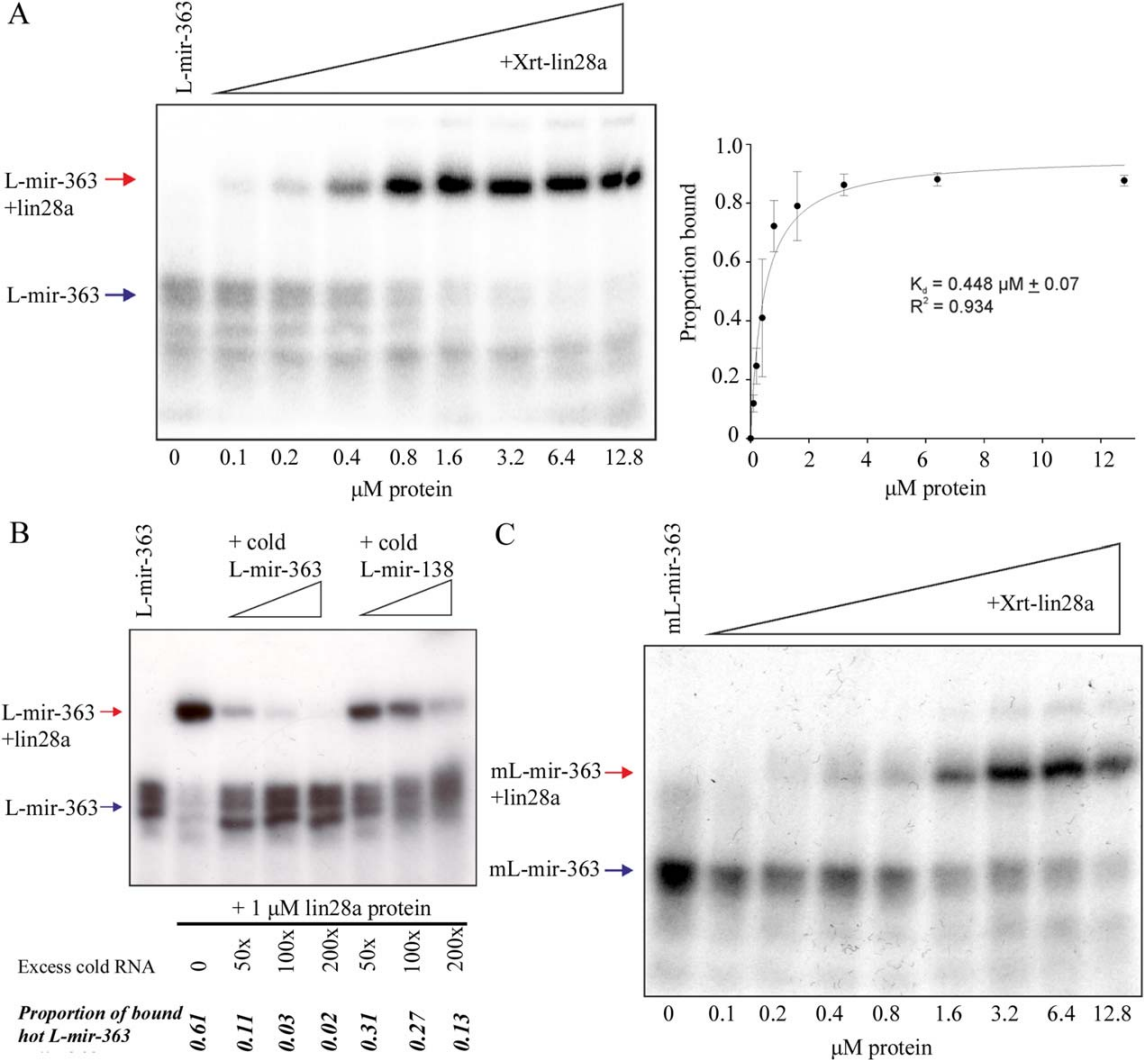
**A**: EMSA performed with ^32^P‐labelled L‐mir‐363 and indicated concentrations of Xrt‐lin28a. Gel shown is representative of n = 3. Band intensities were quantified from three independent experiments and the proportion bound was calculated. Data were fitted by nonlinear regression as described in *Materials and Methods*. Bmax = 0.962. **B**: EMSA performed with ^32^P‐labelled L‐mir‐363 and 1 μM of Xrt‐lin28a (except for RNA only lane). Arrows indicate RNA and lin28a‐RNA complex. Reactions were competed with unlabelled RNA of L‐mir‐363 or L‐mir‐138 in excess levels as indicated. Band intensities were quantified and proportion of RNA bound was calculated. Gel shown is representative of n = 2. Arrows indicate labelled RNA (blue) and lin28‐RNA complex (red). **C**: EMSA performed with ^32^P‐labelled mL‐mir‐363 and indicated concentrations of Xrt‐lin28a. Gel shown is representative of n = 3.

Figure 4A shows that Xrt‐lin28a binds to the terminal loop region of *pre‐mir‐363* (L‐mir‐363) with a similar affinity (K_d_ = 448 nM) to its binding with the *let‐7g* terminal loop. Further evidence for the specific nature of this interaction is demonstrated by the observation that excess cold L‐mir‐363 competes more efficiently for binding of radiolabelled L‐mir‐363 to Xrt‐lin28a, than does the nonrelevant L‐mir‐138 RNA. Thus, in the presence of a 100‐fold excess of cold L‐mir‐363 only 3% radiolabelled L‐mir‐363 remains bound to Xrt‐lin28a, whereas 27% remains bound in the presence of 100‐fold excess of L‐mir‐138 RNA (Fig. 4B).

We next investigated the importance to L‐mir‐363 binding of the GGAG sequence motif present in the mir‐363 terminal loop. A mutant *mir‐363* terminal loop RNA (mL‐mir‐363), in which the GGAG sequence was replaced by a GUAU, was synthesised. The same substitutions have previously been shown to reduce the ability of Lin28 to bind to *let‐7* (Heo et al., 2009). Figure 4C shows that Xrt‐lin28a has a reduced ability to bind mL‐mir‐363 compared with the GGAG containing L‐mir‐363 RNA. Binding of radiolabelled L‐mir‐363 in the presence of 12.8 μM Xrt‐lin28a protein approaches 100%, whereas the binding of the mutant mL‐mir‐363 RNA is reduced to 58%.

### Physical Interaction of Endogenously Translated lin28a Isoforms With pre‐mir‐363

**Figure 5:**
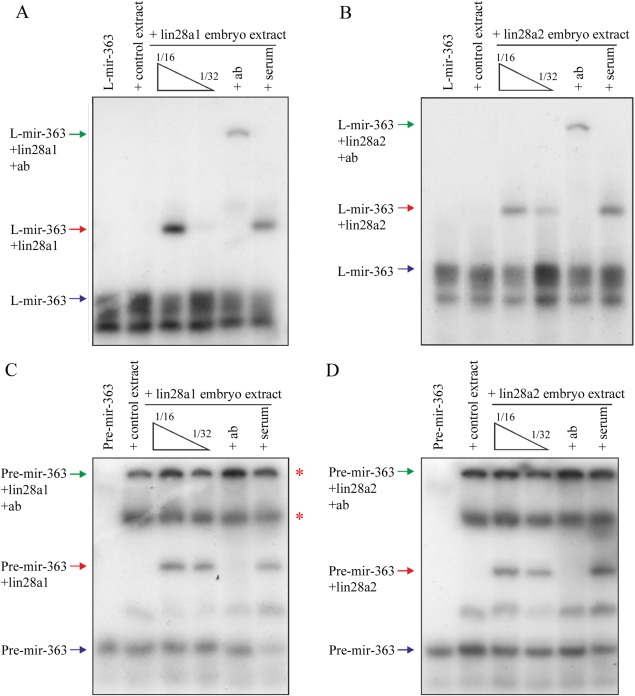
**A,B**: EMSAs performed with ^32^P‐labelled L‐mir‐363 and embryo extract from uninjected controls or embryos injected with 1 ng of either (A) *lin28a1* or (B) *lin28a2*. Embryo extract was used at 1/16 dilution for lanes 2–3, 5–6, and at 1/32 dilution for lower concentration of overexpressing extract in lane 4. Arrows indicate unbound RNA (blue), lin28‐RNA complex (red), and supershift complex of antibody‐lin28‐RNA (green). + Ab = 1/20 dilution α‐lin28a, + ser = 1/20 dilution preimmune bleed serum, both incubated with protein on ice for 20 min before addition of probe. **C,D**: EMSAs performed with ^32^P‐labelled *pre‐mir‐363* and embryo extract from uninjected controls or embryos injected with 1 ng of either (C) *lin28a1* or (D) *lin28a2*. Embryo extract was used at 1/8 dilution for lanes 2–3, 5–6, and at 1/16 dilution for lower concentration of overexpressing extract in lane 4. Arrows indicate unbound RNA (blue), lin28‐RNA complex (red), and supershift complex of antibody‐lin28‐RNA (green). + Ab = 1/20 dilution α‐lin28a, + ser = 1/20 dilution preimmune bleed serum, both incubated with protein on ice for 20 min before addition of probe.

Our experiments using truncated recombinant *Xenopus* lin28a protein have allowed us to investigate the properties and specificity of interactions with the *mir‐363* terminal loop sequence. However, it is important to note that alternative splicing of small 5′ protein coding exons gives rise to two lin28a protein isoforms (lin28a1 and lin28a2) in *Xenopus* (Faas et al., 2013). To investigate binding of these two isoforms to *mir‐363*, we have made use of the ability to overexpress specific proteins from injected synthetic messenger RNAs in the cells of *Xenopus* embryos. Extracts from embryos overexpressing individual isoforms can then be used as a source of full‐length, in vivo translated proteins for use in EMSA assays. Figure 5A and 5B show that extracts from embryos overexpressing either lin28a1 or lin28a2, but not control, nonoverexpressing embryos, contain L‐mir‐363 binding activity. Moreover, we can attribute this binding activity to the overexpressed lin28a proteins because we are able to use an anti‐lin28a antibody, but not a preimmune serum, to deplete the band corresponding to the L‐mir‐363+lin28 complex, giving rise to a higher molecular weight supershifted L‐mir‐363+lin28+Ab complex.

To recapitulate more accurately the binding of the full‐length lin28a isoforms to the native *pre‐mir‐363* structure, we carried out similar binding studies with an in vitro transcribed RNA corresponding to the putative full‐length *pre‐mir‐363*. Again we see that extracts from lin28a overexpressing embryos contain a *pre‐mir‐363* binding activity which can be depleted with a lin28a antibody (Fig. 5C and 5D). Interestingly, all embryo extracts contain at least two additional *pre‐mir‐363* binding activities (asterisks) distinct from that provided by the overexpressed lin28a proteins.

### Temporal and Spatial Expression of mir‐17∼92 and mir‐106a∼363 Clusters in the Embryo

**Figure 6:**
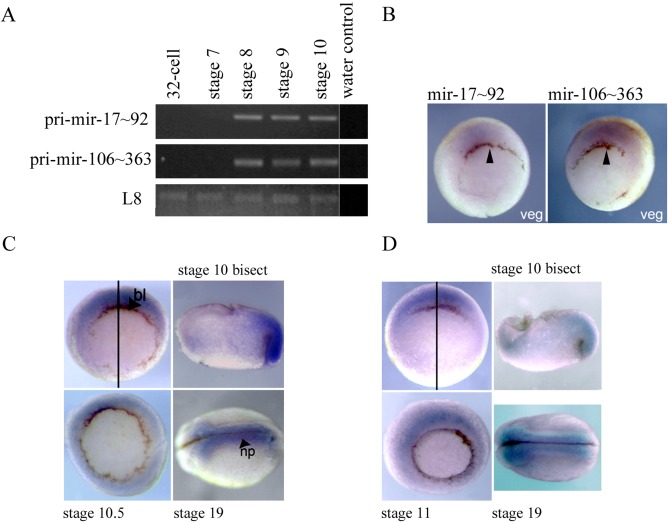
**A**: Developmental time course for expression of *pri‐miR‐17∼92* and *pri‐miR‐106∼363* was undertaken using RT‐PCR. *L8* was used as a loading control. Image is representative of n=2. *miR‐17‐92* = 669 bp, *miR‐106‐363* = 639 bp, *L8* = 435 bp. **B**: In situ hybridisations showing expression of *pri‐miR‐17∼92* and *pri‐miR‐106∼363* RNAs in early development. Vegetal views of early gastrula stage 10.5 embryos, with the dorsal side is to the top. Arrows indicate the dorsal blastopore lip. **C**: In situ hybridisation using an anti‐sense LNA probe showing *mir‐363‐3p* expression in early development. Vegetal views of gastrula stages 10 and 10.5 are shown, with dorsal‐side to the top. An animal to vegetal bisect of a stage 10 embryo is shown with the animal hemisphere to the top and dorsal to the right. A dorsal view of a late neurula stage 19 embryo, anterior to the left. Plane of bisection (black line), dorsal blastopore lip (bl) and neural plate (np) are indicated. **D**: In situ hybridisation using an anti‐sense LNA probe showing *mir‐363‐5p* expression in early development. Vegetal views of gastrula stages 10 and 11 are shown, with dorsal‐side to the top. An animal to vegetal bisect of a stage 10 embryo is shown with the animal hemisphere to the top and dorsal to the right. A dorsal view of a late neurula stage 19 embryo, anterior to the left. Plane of bisection (black line) is indicated.

We have provided evidence for a novel lin28 regulated pathway, involving mir‐17∼92 family miRNAs. However, for the proposed regulatory interactions to be relevant to normal development the components must be expressed in the same cells of the early embryo. We therefore investigated the temporal expression of the primary transcripts from the mir‐17∼92 cluster and mir‐106a∼363 clusters. Primary transcripts for both the mir‐17∼92 and mir‐106a∼363 clusters are initially detected by semi‐quantitative RT‐PCR at mid‐blastula stage 8 (Fig. 6A). This corresponds to the time when the zygotic expression of *lin28a* is initiated (Faas et al., 2013). Figure 6B shows embryos hybridised with RNA probes designed to detect the primary transcripts from the mir‐17∼92 and mir‐106a∼363 clusters in the developing embryo. Highest levels of expression are detected in the dorsal marginal zone of the embryo at early gastrula stage 10.5.

### Spatial Expression of mir‐363‐3p and mir‐363‐5p in the Embryo

Our data provide the strongest evidence for a direct regulatory interaction between amphibian lin28 proteins and the mir‐17∼92 family member, *mir‐363*. Anti‐sense locked nucleic acid (LNA) probes contain modified nucleotides, providing increased sensitivity in detection short RNA sequences, such as miRNAs, and have previously been used to obtain highly specific in situ miRNA localisation in *Xenopus* (Sweetman et al., 2006). Figure 6C and 6D are in situ hybridisation analyses with antisense LNA probes specific for *mir‐363‐3p* and *mir‐363‐5p*. As with the primary cluster transcripts, highest levels of expression are detected in the dorsal marginal zone at the start of gastrulation. Later in development *mir‐363* miRNAs are enriched in the dorsal neural plate. Both miRNAs exhibit expression patterns similar to those previously reported for *lin28*a and *lin28b* (Faas et al., 2013).

## Discussion

### *let‐7* Levels Are Unaffected in Gastrula Stage lin28 Morphants

We have previously shown that lin28 function is required for the very earliest responses of pluripotent cells in the amphibian embryo to germ layer specifying growth factors. For example, levels of mesoderm lineage specific marker genes such as *brachyury, myoD*, and *chordin* are significantly reduced in early gastrula stage compound lin28 morphants (Faas et al., 2013). Furthermore, we found that levels of mature *let‐7a, f*, and *g* miRNAs are not significantly affected in gastrula stage lin28 morphants, leading to the proposition that, during the very earliest stages of amphibian development, lin28 proteins have functions independent of regulating *let‐7* biogenesis. Here we complement and extend this analysis using miRNA microarray‐based assays, and again we detect no significant changes in the levels of any of the *let‐7* family miRNAs represented on either microarray platform (*let7a, b, c, e, f, g*, and *i*) in gastrula stage lin28 morphants. However, we note that expression levels of *let‐7* miRNAs are generally low and some family members are not detected at all (data not shown). Similar conclusions were drawn in a zebrafish lin‐28 knockdown study, where no significant changes in *let‐7* expression were found in morphants at 5 hpf (Ouchi et al., 2014). However, in *Xenopus* and zebrafish, increased levels of *let‐7* miRNAs are detected in lin28 morphants during later development, postneurula stage 22 and 28 hpf, respectively, indicating that in both species there is an early *let‐7*‐independent, and a late *let‐7*‐dependent role for lin28 proteins (Faas et al., 2013; Ouchi et al., 2014).

### 17∼92 and 106∼363 Cluster miRNAs Are Down‐Regulated in Gastrula Stage lin28 Morphants

Our array analysis of morphants indicates no significant up‐regulation of any miRNAs. However, several miRNAs are shown to be down‐regulated, indicating a novel role for *Xenopus* lin28 proteins as positive regulators of miRNA abundance. A notable feature of this down‐regulated group is the enrichment for members of the mir‐17∼92 cluster (*mir‐17‐5p, mir‐19a*, and *mir‐20a*) and the paralogous mir‐106∼363 cluster (*mir‐19b, mir‐20b, mir‐363‐5p*, and *mir‐363‐3p*). These data suggest a novel regulatory interaction, where lin28 proteins act as positive regulators of 17∼92 family miRNAs.

However, we find that increasing levels of lin28 proteins does not lead to a significant complementary up‐regulation of 17∼92 family miRNA abundance. This suggests that endogenous levels of lin28 proteins are sufficient to allow maximal production of mature 17∼92 family miRNAs. Thus, lin28 levels only become limiting following knockdown. This is supported by the observations that lin28 morphants exhibit a strong phenotype, which can be rescued by lin28 mRNA injection (Faas et al., 2013), whereas embryos injected with lin28 mRNA alone develop normally (Fig. 2B).

The paralogous mir‐17∼92 and mir‐106∼363 clusters each code for six miRNAs, which have been highly conserved during vertebrate evolution. The mir‐17∼92 cluster, in particular, has attracted a great deal of interest in recent years in relation to normal cellular function and its oncogenic potential (reviewed Mendell, 2008; Olive et al., 2010; Mogilyansky and Rigoutsos, 2013). Studies in mammals indicate that 17∼92 cluster miRNA expression is high in embryonic cells, and is associated with the pluripotent state. 17∼92 miRNA expression has been proposed to be part of the miRNA signature of human embryonic and induced pluripotent stem cells (Wilson et al., 2009). Mutations in the 17∼92 cluster are associated with Feingold syndrome in humans, which is characterised by skeletal dysplasia (Marcelis et al., 2008). Deletion of the 17∼92 locus in mice also leads to abnormal skeletal development. The phenotype of 17∼92 null mice indicates additional roles in embryonic growth and morphogenesis of the heart and lungs (Ventura et al., 2008; de Pontual et al., 2011).

The mir‐106∼363 cluster is less well studied. It has been reported that in mammals miRNAs from this cluster are not widely expressed and development of mice lacking the mir‐106∼363 cluster is apparently normal (Ventura et al., 2008). In contrast, we find that all six 106∼363 cluster miRNAs are expressed by the early amphibian embryo (Supp. Table S1). Of particular interest to the present study are *mir‐363‐5p* and *mir‐363‐3p*, which are derived from a common *mir‐363* precursor RNA. Quantitative analysis of miRNA abundance in morphants indicate that, of the miRNAs analysed, *mir‐363‐5p* and *mir‐363‐3p* are most sensitive to lin28 inhibition.

### Lin28 Proteins Bind the Terminal Loop Region of Multiple miRNAs

Lin28 proteins physically interact with primary and precursor *let‐7* miRNAs (reviewed Mayr and Heinemann, 2013). This interaction is, in part, at least, mediated by GGAG or GGAG‐related sequences in the terminal loop of *pre‐let‐7* miRNAs, and binding provides the basis for the negative regulatory effects of lin28 on the biogenesis of mature *let‐7* miRNAs (Heo et al., 2009; Mayr and Heinemann, 2013). It is tempting to speculate that the physical interaction of lin28 proteins with 17∼92 family RNAs might also be required for the positive regulatory interaction that we report here. It has been previously reported that LIN28A is able to bind to the human mir‐363 precursor by means of a GGAG motif in its terminal loop (Heo et al., 2009). This GGAG motif is conserved in *Xenopus pre‐mir‐363* and both recombinant and endogenously overexpressed lin28a physically interact with the terminal loop sequence of *Xenopus pre‐mir‐363*. The affinity of this interaction is comparable with the observed for the interaction between lin28a and the *let‐7g* terminal loop. Furthermore, we find that mutation of GGAG sequence in the *mir‐363* terminal loop reduces the affinity of this interaction. In keeping with the notion that lin28 proteins and mir‐17∼92 miRNAs physically interact, we find that both *mir‐363* miRNAs are expressed in similar domains to *lin28a* and *lin28b* in the presumptive mesoderm of gastrula stage *Xenopus* embryos (Faas et al., 2013).

Lin28 proteins inhibit the biogenesis of *let‐7* miRNAs using multiple mechanisms. One reported mechanism requires LIN28A dependent recruitment of the Tut4 enzyme to a *pre‐let‐7* containing complex and subsequent Tut4 mediated polyuridylation and inactivation of *pre‐let7* miRNAs (Heo et al., 2008, 2009). Several other human miRNAs have been shown to contain a terminal loop GGAG motif and are bound by LIN28A; however, this association does not always lead to Tut4 mediated polyuridylation and destabilisation (Heo et al., 2009). Thus, Lin28 binding to a GGAG motif in the terminal loop can have different consequences, depending on the target miRNAs involved.

At present, we do not know the mechanism by which amphibian lin28 proteins promote the expression of 17∼92 family miRNAs in the early embryo. Indeed, our data do not rule out the possibility of lin28 proteins, indirectly or directly, regulating 17∼92 miRNA expression by multiple mechanisms. However, an attractive hypothesis is that the binding of lin28 to the terminal loop region of the *pre‐mir‐363* sequence within the 106∼363 polycistron somehow promotes subsequent processing to the precursor and mature miRNAs derived from the primary transcript. In regard to the related 17∼92 polycistron, we have not yet investigated physical interactions with lin28 proteins. We have not identified GGAG‐like sequences in the terminal loop region of 17∼92 polycistron derived pre‐miRNAs; however, there are multiple GGAG motifs present within the intergenic regions of the polycistronic primary transcript (data not shown). It will be interesting to determine whether these motifs can act as binding sites for lin28 proteins.

### Lin28, let‐7 Family, and 17∼92 Family; Key Components of a Pluripotency Network

Lin28 expression is associated with pluripotent mammalian stem cells in culture and pluripotent cells in the early amphibian embryo that respond to the earliest lineage specifying growth factor signals. A key function of Lin28 proteins in mammalian stem cells is to inhibit the biogenesis of *let‐7* miRNAs. During differentiation of stem cells, lin28 levels fall and levels of biologically active *let‐7* miRNAs rise (Viswanathan et al., 2008; Viswanathan and Daley, 2010). While a role for lin28 regulating *let‐7* biogenesis in postneurula stage amphibian embryos is supported, there is no evidence that this function is important in very early development, perhaps because transcription of *let‐7* miRNAs is low. In contrast to *let‐7* miRNAs, elevated expression of 17∼92 family miRNAs is associated with the pluripotent state. It is tempting to speculate that in some pluripotent cell populations lin28 proteins might play a dual role in inhibiting *let‐7* and promoting 17∼92 family expression. We note that similar dual, opposite effects on the regulation of *let‐7* and 17∼92 miRNAs have also been reported for the hnRNPA1 RNA binding protein, which like Lin28 inhibits *let‐7* biogenesis, but promotes the biogenesis of the 17∼92 cluster miRNA, *mir‐18a* (Guil and Caceres, 2007; Michlewski and Caceres, 2010).

Taken together, our results suggest a novel regulatory function for lin28 proteins in the pluripotent cells of the early amphibian embryo, in which lin28 proteins positively regulate levels of mature 17∼92/106∼363 cluster miRNAs in the early embryo, which contrasts with their activity of negatively regulating mature *let‐7* miRNA levels in mammalian stem cell populations.

## Experimental Procedures

### Embryo Methods

*Xenopus tropicalis* embryos were produced as previously described (Khokha et al., 2005; Winterbottom et al., 2010). Embryos were injected at two‐ or four‐cell stage and cultured at 22 ºC.

Samples for miRNA analysis were isolated using the miRVana miRNA isolation kit (Applied Biosystems). The protocol was carried out according to manufacturer's instructions with the modification that after lysis samples were centrifuged for 10 min at 4 °C and supernatant removed to a fresh tube.

### Western Bot Analysis

Western blots were carried out as previously described, using affinity‐purified *X. tropicalis* anti‐lin28 antisera raised by inoculation of peptides corresponding to the C‐terminal sequences of *X. tropicalis* lin28a1/a2 (EEQPISEEQELIPETME) or lin28b (SRKGPSVQKRKKT) proteins (Faas et al., 2013).

### Knockdown of lin28a and lin28b

Compound knockdown of lin28a1+a2+b was accomplished using a total of 25 ng per embryo of a mixture containing 10 ng lin28a1 + 10 ng lin28a2 + 5 ng lin28b AMOs (Gene Tools, LLC), as previously described (Faas et al., 2013). Injections were carried out into all cells at either the two‐ or four‐cell stage, with a maximum of 10 nl/embryo. Injections were targeted to the marginal zone.

### Overexpression of *lin28a* and *lin28b*

The coding regions of *lin28a1, lin28a2*, and *lin28b* were PCR amplified and sub‐cloned into the Cs2 + mRNA transcription vector. mRNA synthesis was as previously described (Branney et al., 2009). All cells were injected at the two‐ or four‐cell stage, with a total of 1 ng/embryo of each mRNA. Injections were targeted to the marginal zone.

### Affymetrix miRNA Array Analysis

RNA was isolated from control embryos and knockdown as described above at early gastrula stage 10.5. The quality of the RNA was verified using the Agilent 2011 Bioanalyzer (Agilent). Samples were processed in the University of York, Department of Biology Technology Facility. One microgram samples were processed in the University of York, Department of Biology Technology Facility. RNA was labelled using HSR FlashTag Biotin RNA labelling kit (Genisphere) according to manufacturer's instructions, which included the addition of spike‐in RNA controls to act as a method control. Samples were then hybridised to Genechips miRNA 2.0 (Affymetrix) overnight, and washed on a Fluidics Station 450 (Affymetrix), all carried out according to manufacturer's instructions. Scanning of the chips was carried out using an Affymetrix Genechip Scanner. CEL files were processed using Affymetrix QC tools software to provide background detection and quantile normalisation with a final median polish and log transformation. *Xenopus* feature data were extracted and statistical comparisons undertaken using a paired, two‐tail Student's *t*‐test. The complete triplicate summarization data set for the *Xenopus* features are shown in Supplementary Table S1. These data have been deposited in the ArrayExpress Archive (https://www.ebi.ac.uk/arrayexpress/) with accession number E‐MTAB‐3936.

### Exiqon miRNA Array Analysis

Compound knockdown of lin28a1+a2+b was carried out as described above. Control embryos were injected with 30 ng of a standard control MO. RNA was isolated from experimental embryos at late gastrula stage 13. Quality control, sample processing, and preliminary data processing, including normalization were undertaken as a service by Exiqon A.S. Expression levels were calculated as fold changes relative to a mixed stage reference RNA sample. *Xenopus* feature data were extracted and statistical comparisons were undertaken using a paired, 2‐tail Student's *t*‐test. The median log ratios for the triplicate *Xenopus* data set are shown in Supplementary Table S2. These data have been deposited in the ArrayExpress Archive (https://www.ebi.ac.uk/arrayexpress/) with accession number E‐MTAB‐3939.

### Semi‐quantitative PCR Analysis of miRNA Cluster Primary Transcript Abundance

Total RNA was extracted using TRI reagent (Sigma) according to the manufacturer's instructions. An additional precipitation step was undertaken using 7.5 M LiCl and 0.05 M EDTA at ‐80 °C overnight. cDNA was synthesised from total RNA using 1 μg RNA random hexamers (Invitrogen) and SuperScript II Reverse Transcriptase (Invitrogen) according to manufacturer's instructions. cDNA was diluted 1/5 for use in RT‐PCR reactions using PCR Master Mix (Promega). Primer sequences are shown below.

**Table:** 

L8 forward:	GGGCTRTCGACTTYGCTGAA
L8 reverse:	ATACGACCACCWCCAGCAAC
miR‐17∼92 cluster forward:	TGCAGTGAAGGCACTTGTAG
miR‐17∼92 cluster reverse:	TAAACAGGCCGGGACAAG
mir‐106a∼363 cluster forward:	TGCTGGACACCTGTACT
mir‐106a∼363 cluster reverse:	TTCTGCGGTTTACAGATGGA

### miRNA qRT‐PCR

Samples to be used for miRNA analysis were isolated using the miRVana miRNA isolation kit (Applied Biosystems) as described above.cDNA was synthesised from 10 ng RNA/RT reaction with miRNA‐specific primers for TaqMan assays (Applied Biosystems) using the TaqMan MicroRNA Reverse Transcription Kit (Applied Biosystems) as manufacturer's instructions.qRT‐PCR was carried out using TaqMan Universal Master Mix II (Applied Biosystems) with Taqman miRNA probes (Applied Biosystems) according to manufacturer's instructions. All reactions were performed in quadruplicate per sample on an ABI Prism 7000 detection system (Applied Biosystems) with thermal cycling at 95 °C for 10 min, followed by 40 cycles of 95 °C for 15 sec and 60 °C for 1 min. Gene expression levels were normalised to U6 snRNA using the 2^‐ΔΔCt^ method. Preliminary experiments had shown that U6 snRNA was a suitable control for this purpose (data not shown). Assays used were: hsa‐miR‐19b, hsa‐miR‐20b, hsa‐miR‐363#, hsa‐miR‐363, hsa‐miR‐18b, hsa‐let‐7a, hsa‐let‐7f, custom xtr‐mir‐106a (Applied Biosystems). It is important to note that the inclusion of stem loop structures in the primers used in the miRNA specific cDNA syntheses allow for the detection of mature, biologically active miRNAs.

### Whole‐Mount In Situ Hybridisation for miRNA Cluster Primary Transcripts

To generate whole‐mount in situ hybridisation probes for the miR‐17∼92 and mir‐106a∼363 clusters, cDNAs corresponding to sections of the miR‐17∼92 and mir‐106a∼363 primary transcripts were cloned in the pGEM®‐T Easy vector following PCR amplification using *X. tropicalis* genomic DNA as template and the following primers.

**Table:** 

mir‐17∼92 cluster forward:	TGCAGTGAAGGCACTTGTAG
mir‐17∼92 cluster reverse:	TAAACAGGCCGGGACAAG
mir‐106a∼363 cluster forward:	TGCTGGACACCTGTACT
mir‐106a∼363 cluster forward:	TTCTGCGGTTTACAGATGGA

Digoxigenin (DIG) ‐labelled antisense in situ probes were transcribed and in situ hybridisation was carried out as previously described (Harland, 1991) with slight modification (Reece‐Hoyes et al., 2002).

### Whole‐Mount In Situ Hybridisation for miRNA

Probes used were 5′‐DIG labelled LNA miRNA detection probes (Exiqon), named “hsa‐miR‐363‐3p,” and “xtr‐miR‐363*.” Protocol was carried out as described previously (Sweetman, 2011), with modifications advised by Grant Wheeler (University of East Anglia, UK, personal communication). Probes were preabsorbed six times by hybridising with the probe overnight against stage 35 embryos. Colour development was with NBT/BCIP substrate. When signal began to develop, embryos were washed at 4 °C overnight and subjected to repeat cycles of colour reaction and washes until a strong specific signal. Embryos were then fixed and bleached with hydrogen peroxide to remove pigment before photography.

### Recombinant Lin28 Protein Production

Relevant coding sequences were cloned into the pET28a expression vector. Recombinant Lin28 proteins were expressed overnight at 16 °C in B834 *E. coli* cells grown in LB media, following induction with 1 mM IPTG. Cells were harvested by centrifugation and pellets resuspended in a solution containing either 50 mM sodium phosphate pH 7.8, 250 mM NaCl 1 mM DTT, 20 mM imidazole, and 10% w/v glycerol (rt‐LIN28A); or 50 mM Tris HCl pH 7.5, 500 mM NaCl, 0.5 mM DTT, 20 mM imidazole, and 10% w/v sucrose (Xrt‐lin28a). The resuspension solution was supplemented with 0.5 μg/ml leupeptin, 0.7 μg/mL pepstatin, and 1 mM AEBSF protease inhibitors. Cells were lysed by sonication and the lysate applied to a 5 ml HisTrap column (GE Healthcare). Bound protein was eluted using a linear imidazole gradient (20–500 mM). Fractions containing Lin28 were analysed by sodium dodecyl sulfate‐polyacrylamide gel electrophoresis (SDS‐PAGE), pooled, concentrated, and applied to an S200 gel filtration column (GE Healthcare) in a running buffer consisting of either 20 mM Tris pH 7.5, 150 mM NaCl, 1 mM DTT, 10% w/v glycerol (rt‐LIN28A), or 10 mM Tris pH 7.5, 150 mM NaCl, 2 mM DTT, and 10% w/v sucrose (Xrt‐lin28a). Eluting fractions containing Lin28 were analyzed by SDS‐PAGE, pooled and concentrated, before being flash frozen in liquid N_2_ and stored at ‐80 °C. Proteins were produced as N‐terminal fusions with the sequence, MGSSHHHHHHSSGLVPRGSHM, containing a His‐tag and thrombin digest site. In the case of the human rt‐Lin28a, the N‐terminal His‐tag was removed by thrombin digest before gel filtration.

### Full‐Length Human LIN28A Protein

MGSVSNQQFAGGCAKAAEEAPEEAPEDAARAADEPQLLHGAGIC KWFNVRMGFGFLSMTARAGVALDPPVDVFVHQSKLHMEGFRSL KEGEAVEFTFKKSAKGLESIRVTGPGGVFCIGSERRPKGKSMQKRR SKGDRCYNCGGLDHHAKECKLPPQPKKCHFCQSISHMVASCPLKA QQGPSAQGKPTYFREEEEEIHSPTLLPEAQN

### N‐ and C‐Terminally Truncated Human LIN28A Protein (rt‐LIN28A), Residues 37‐180

LLHGAGICKWFNVRMGFGFLSMTARAGVALDPPVDVFVHQSKLH MEGFRSLKEGEAVEFTFKKSAKGLESIRVTGPGGVFCIGSERRPKG KSMQKRRSKGDRCYNCGGLDHHAKECKLPPQPKKCHFCQSISHMV ASCPLKAQQ

### N‐ and C‐Terminally Truncated *Xenopus* lin28a Protein (Xrt‐lin28a), Residues 34‐177

**MGSSHHHHHHSSGLVPRGSHM**GSGVCKWFNVRMGFGFLTMTK KEGTDLETPVDVFVHQSKLHMEGFRSLKEGESVEFTFKKSSKGLEST RVTGPGGAPCIGSERRPKVKGQQKRRQKGDRCYNCGGLDHHAKEC KLPPQPKKCHFCQSPNHMVAQCPAKASQAAN. (Leader containing the His‐tag and thrombin cleavage site is indicated in bold.)

### RNA EMSAs

Pre‐cursor *mir‐363* RNA was synthesised in vitro. DNA templates for *pre‐mir‐363* were produced by PCR, to include an SP6 RNA polymerase promoter at the beginning of the sequence, using a plasmid containing the *Xenopus* mir‐106a‐363 as template and the following primers. RNA was synthesised using SP6 Megascript kit (Ambion) according to manufacturer's instructions.

*Pre‐mir‐363*

**Table:** 

Forward: ATTTAGGTGACACTATAGGGCTGAGGTAGTTGTTT	Reverse: TAGGCAAGGCAGTGGCCTGTACAG

RNA oligonucleotides used in RNA mobility shift assays were synthesised by Dharmacon.

L‐mir‐138

UUGUGAAUCAGGCCGUGACCACUCAGAAAACGGCUACUUCA CAAC

L‐mir‐363

UGCAAUUUUAUUUAGUUUGGUAGGAGAAAAAUUGCmL‐mir‐363

UGCAAUUUUAUUUAGUUUGGUAUGAUAAAAAUUGC

RNA oligonucleotides and mir‐RNA precursors were radioactively labelled with ^32^P ATP using the KinaseMax kit (Ambion) according to manufacturer's instructions.

Recombinant protein EMSAs were performed with the proteins described above. For embryo extract EMSAs, uninjected *X. laevis* controls embryos and embryos injected with 1 ng *lin28a1, lin28a2*, or *lin28b* mRNA (Faas et al., 2013) were lysed in 50 mM Tris‐HCl pH 7.9, 25% glycerol, 50 mM KCl, 2 mM DTT, 0.1 mM EDTA, 1/100 Protease inhibitor cocktail III [Calbiochem]) at 10 μl/embryo. Lysates were cleared by centrifugation and extracts were diluted as required in the lysis buffer.

Binding reactions were carried out as described previously (Piskounova et al., 2008). Labelled RNA probes were incubated with protein in binding buffer (60 mM KCl, 10 mM HEPES, pH 7.6, 3 mM MgCl_2_, 5% glycerol, 1 mM DTT, 5 μg/μl heparin [Sigma], and 150 ng yeast total RNA competitor [Ambion]) for 30 min at room temperature.

The custom anti‐lin28a antibody (Enzo Life Sciences (UK) Ltd), used for the embryo extract supershift assays, has been previously described (Faas et al., 2013) and was used at 1/20 dilution per binding reaction, with 1/20 dilution preimmune bleed used as a serum control. 20 units of RNAsin (Promega) were added per binding. Antibody was preincubated with protein and binding buffer for 20 min on ice, before labelled probe was added for a further 20 min at room temperature.

Samples were run on a 10% native polyacrylamide gel. Gels were dried and exposed either to a Phosphor Screen (GE Healthcare) and were scanned, processed and analysed using a Bio‐Rad Molecular FX Imager and Quantity One software (Bio‐Rad); or exposed to Hyperfilm ECL film (Amersham) and films analysed using Image J. The proportion of RNA bound at each protein concentration was calculated, and the K_d_ determined by nonlinear regression using the SigmaPlot software package, with the equation:

$$\text{Proportion}\;\text{bound}=\frac{B_{\max}\left[\text{lin}28\right]}{K_{d}+\left[\text{lin}28\right]}$$

## Supporting information

Additional supporting information may be found in the online version of this article

Supporting Information Table 1.

Supporting Information Table 2.
